# Prolapsus Uteri with Ulceration

**Published:** 1861-08

**Authors:** Daniel D. Waite

**Affiliations:** Chicago, Illinois


ARTICLE II.

### PROLAPSUS UTERI WITH ULCERATION.

A Paper by DANIEL D. WAITE, M. D., Chicago, Illinois.

Read before the Chicago Medical Society, June, 1861.

Do not suppose that I intend to make an essay upon this subject. I intend no such thing. All that I propose at this time is, to relate a case that I deem of some interest, which lately came under my notice, accompanied with some preliminary remarks, and such reflections and practical observations as would naturally arise from the case.

Under the term prolapsus, I think should be included all descent of the uterus of sufficient extent to demand the attention of the physician. Relaxation or weakness, prolapsus or procedintia, according to the books, are but so many degrees of descent; and these stages of the difficulty may be divided into three, six, or a dozen, according to the ingenuity of him who undertakes the task. But it is doubtful if they serve any useful purpose, as the remedy to be applied obviously depends upon the stage of the prolapsus, without reference to a name, and this must be learned in all cases by actual examination.

Probably the remark will be sustained by facts, that of all chronic derangements of the womb, prolapsus is the most common. It is probable that not one case in ten of those that become troublesome, can come to the actual knowledge of the physician. We had almost said that one-half of the child-bearing women of the country are in the habit of uttering such complaints, as would justify the conclusion that the womb was not in its normal position. The cause of this great prevalence of the complaint in this country, beyond that of most nations, has been, and still is, a subject of interesting enquiry, but cannot be examined on this occasion.

The physiological changes upon which prolapsus depends, have been a subject of much dispute. While some contend that the principal changes, or proximate causes, are weakness, relaxation and stretching of the ligaments, others as earnestly contend that the ligaments do not figure in the case at all. Even our own Dewees, doubted if the ligaments had anything to do in sustaining the womb in place. Following out this idea, we should appear to be led to the logician's *reductio ad absurdum;* for no other use can be assigned the ligaments; and if they do not aid in sustaining the womb, they must have been formed for no purpose—which is absurd. Is a machine, so wonderfully and exquisitely wrought as the human system, made up, in part, of useless appendages? Doubtless, in my mind, the ligaments contribute, in conjunction with other parts, in keeping the womb *in situ.* But when the other sustaining parts give way, the ligaments, not being sufficient in themselves, also give way, and allow the womb to descend by their extension. While I concede that the vagina, the bladder, the rectum, and the muscles of the pelvis, all aid in bearing up the womb, I am nevertheless of the opinion that the perineum is the great sustaining part, constituting, as it does, the base of the pelvis.

The case I am about to relate, would seem to sustain this opinion. About eighteen months ago, I was consulted in relation to a falling of the womb, by a lady of fifty-five, naturally strong, robust and healthy, and accustomed to moderate or ordinary labor. I learned from her history, that for the last eight or ten years she had been troubled with a pressure of the womb downwards. The difficulty had been constantly increasing, till within the last three months she had been unable to be on her feet more than an hour or two at a time without having the organ come entirely into the world, as she expressed it; and that she was in the habit, though in great distress in her back and loins, of travelling miles about the city in that condition. Although I knew that parallel cases existed in the books, yet never in my life having seen as bad a case as she represented her's to be, I confess I was somewhat incredulous, supposing there must be some mistake about the case; but upon examination I found it to have been truly stated. The whole organ, about once and a half its natural size, or that much larger than an ordinary sized womb, was entirely outside the body, and on a line with the same, except that the os was drawn a little back, or towards the perineum. (This has always been its position when without the body.) Although perfectly procidented, it was held firmly up to the vulva, and it was a little difficult to pass the finger over the fundus. Except the enlargement, and a slightly purple hue, the uterus appeared healthy. It was so easily replaced that I was suspicious the external orifice had been injured. I enquired if the external parts had ever been lacerated; she answered no; but upon reflection, said she was told the parts were injured at the birth of her first child, in early life; but that she supposed they had been healed. She had borne two children. Considering the case a formidable one, Professor Byford was called in consultation. Upon further examination, we ascertained that the perineum had been narrowed to about one-half its natural breadth. The vagina was obviously very much enlarged, which I attributed mainly to its expansion by the enclosed womb which, it so long carried in a probably enlarged condition. But here the question arises,—did the relaxed vagina allow the descent of the uterus, existing prior to the descent, or was its relaxation caused by the continued pressure of the organ upon its parietes? There are no data upon which a confident opinion can be given, but I strongly incline to the latter supposition. If we consider the purposes which the vagina is destined to fulfill; its great capacity for expansion, and its frail structure, I think we may arrive at the conclusion that it does not *per se* afford much sustaining power to the womb, only as it fills a certain space between the womb and the perineum, upon which it itself rests. In numerous cases, the womb will be found prolapsed into the vagina, and resting upon the perineum. This has been a common occurrence in my own practice, from which I must in a great measure draw my conclusions; and I presume, from what I have learned, that it is not uncommon anywhere. But it seems that the perineum and sphincter vagina, will, in ordinary cases, long sustain the womb without allowing it to protrude. In the case under consideration, the woman had tried a variety of pessaries, without much benefit, as the pendant womb would soon press them out, and for the very plain reason that they had no sufficient floor to rest upon.

Professor Byford recommended the India rubber inflation pessary, which was procured. But this, although inflated to the size of a man's fist, would, after a short time upon the feet, be forced through the os externum. The Doctor carefully measured the womb, outside and in, from which measurement the conclusion as to its engorgement and enlargement was arrived at. With the advice of close and firm bandaging, we left her, in the belief that the case was probably incurable, except by an operation that would diminish the size and capacity of the vagina. This she was not inclined to submit to at the time, nor, indeed, were we prepared strongly to advise it. I saw her frequently during the ensuing year, trying to keep house, and, of course, much upon her feet, carrying the womb a large share of the time, when not in bed, between the thighs. It is proper to say, that she had an almost constant leaking of the urine, which soon wet the bandages and clothing, rendering them not only uncomfortable, but offensive. She always said she got along very badly, but as well as she could. She said she sometimes felt as though she should "drop down in her tracks," such was the distress in her back and loins, and occasionally across her body. She is naturally a woman of great fortitude and courage, but now becoming nervous and irritable.

Three months ago, she applied again. She said she was much worse; and could not live so any longer. She said blood and matter in considerable quantities were constantly discharged; that she was getting very weak, and that it hurt her very much to put up the womb. Examining, I found the mouth of the womb extensively ulcerated, and the whole organ irritated and more enlarged. One side of the os, including nearly one-half of the ring, was entirely eaten away, the ulcer extending inwardly as far as could be seen, and outward and upward more than an inch. From several other points at and near the os, and from the inside, matter exuded on pressure. If "seeing is believing," in this case seeing was knowing. No imperfect speculum knowledge was needed. Here we had the naked fact; and naked enough too. After thoroughly washing the parts, I applied the hard lunar caustic to the ulcerated portions, burning it severely, and passing it slightly over the lower half of the organ. This I repeated every other day, for five or six times. Still on discontinuing the application a week, the ulcers resumed nearly the same appearance, and all the while discharging largely a thin, greenish-white matter. Somewhat discouraged, and fearing from the irregular and somewhat jagged appearance, some specific tendency in the ulcers, Dr. O. Smith was consulted. On examination, the Dr. although somewhat suspicious of the ulcers, thought they might probably be cured; and for that purpose, recommended a strong solution of the per-sulphate of iron. This application I used for about a week, producing a favorable appearance, but not seeming to have a sovereign effect. Ascertaining that I could not obtain a strong tincture of this article, I obtained, at the suggestion of the apothecary, a concentrated tincture of the per-chloride of iron. This, from Reed's, was rather more escharotic than desirable, and was reduced by one-half water. This application with a large camel's hair pencil, turned the parts a light yellow, and seemed to act as a powerful astringent, rendering the muscle hard and firm to the touch. After being applied every other day, thoroughly, for five or six times, the discharges ceased. The application was continued in this way about two weeks, the ulcers being then about half healed, and the womb firmer and smaller. The action of the chloride was not confined to the ulcerated portions, but passed freely over a large share of the body of the womb. From the period of my commencing with the ulcers, the iodide of potassium had been exhibited in pretty free doses, which, by the advice of Dr. Smith, was carried as high as forty grains per day. Three weeks after the last application, I found the parts entirely healed, the womb looking healthy, and, as near as I could judge, of the natural size.

Ashwell says: "The womb may be irritated into ulceration by pessaries and other local causes." And he adds, "this may occur with much bleeding in case of entire procidence. We all know the varying depth and extent of such ulcers, and how extremely difficult they are to heal." It seems pretty clear that in this case the womb had been goaded into ulceration by the friction of external irritants.

It may be worthy of notice that no application to the uterus produced any pain, or apparent sensation, unless carried within the os. Even when thoroughly burned with the nitrate of silver not the least complaint was made. A solution of the nitrate, or the tincture carried a little within the neck, produced an immediate start, followed by pain in the back.

A *T* bandage, protected by oiled silk, is now worn, and the womb in this way is kept within the vulva, except as it will occasionally slip out. Since the reduction of its size, it seems much easier to keep it within the body. And I think the tincture has had an astringent effect upon the vagina, and perhaps upon the ligaments. Certain it is that the womb is more easily retained inside now than formerly. She is quite thankful for her more comfortable condition, and unless she gets worse, will not be likely to submit to an operation, if advised.

Query: What further can be done for this woman? Is the operation of excising a portion of the vagina safe and advisable?

Perhaps I should say that I have vainly endeavored to confine this patient to a horizontal position while a cure was attempted. For a few days, while at the worst, she was on her couch, say one half of the time. But at the present time, one month after the cure of the uterus, she is keeping an apple and candy shop, and although complaining severely of her back and hips, she is almost continually on her feet, except during the usual hours of rest.

ADDENDA.—The above communication was read before the Chicago Medical Society on the third Friday in June. Two weeks after this date I was again consulted; the woman saying that there was again some discharge of blood and matter. On examination, I found a small ulcer on the location of the former one, and some blood escaping from the os. The os was also somewhat swollen from irritation. Three or four applications of the per-chloride healed it perfectly, and stopped the discharge of blood. The womb now again appears healthy, and does not as readily escape from the body.

D. D. W.

CHICAGO, July 9, 1861.

---

ARTICLE III.

